# The Treatment of Albuminuria in Pregnancy

**Published:** 1902-01-04

**Authors:** 


### THE TREATMENT OF ALBUMINURIA IN PREGNANCY.

In describing the line of treatment to be adopted when albuminuria is present in a case of pregnancy, Dr. Berry Hart[1] says that in many cases milk diet, rest in bed, attention to the bowels, and an occasional hot bath, are all that is necessary, and that cases treated in this way seldom go on to the more serious stages. "When, however, the case becomes worse, headache or eye symptoms supervening, with the urine still albuminous, induction of labour is indicated and its non-performance in such a case is a serious error." The important and urgent complication however is eclampsia. In dealing with this condition treatment was at one time, and still is with some, heroic and violent; all of which Dr. Berry Hart thinks is a great mistake. It is the dying legacy of the uræmic theory. The administration of powerful drugs, the use of pilocarpin, and venesection, ought to be abandoned. They have no exact basis of fact to support them, and they are apt to undermine the strength of the patient. Dr. Berry Hart recommends that, after thoroughly examining the patient in regard to possible complications, we should begin with a hypodermic injection of morphia, $\frac{1}{3}$ to $\frac{1}{2}$ grain, and repeat it in two or three hours if necessary, but this time giving only $\frac{1}{4}$ grain. Not more than 1 or 2 grains should be given in the twenty-four hours. This use of morphia has long been known in Germany, but up to recently such a use of the drug would have been considered a serious mistake in England, although in old obstetric works laudanum was recommended in the treatment of this condition. Chloroform by inhalation and chloral by rectum are excellent remedies; chloroform being unsurpassed for rapidity of action, but of course requiring the doctor to be constantly present, whereas if morphia act well the patient may be left for a time. The effect of morphia is much more lasting than that of chloroform. If there be coma morphia is generally withheld, although there seems no theoretical objection to its use. The next thing is to give a large saline injection by the bowel, and then, if there be no improvement, a submammary transfusion of saline solution. This should run to the amount of two to three pints, and in a bad case the saline may be injected into a vein. The hot pack may also be found useful. There is no objection to one smart purge, but pilocarpin certainly should not be used. It may increase lung œdema and cause a distressing accumulation of saliva. The progress of the labour may be accelerated, but unless in very extreme cases there is no call for the rapid method of Dührssen or for Cæsarean section. Pathologists have not yet been able to ascertain the nature of the poison by which eclampsia is caused—if indeed it is so caused—but at least we have learned that the kidney occupies only a secondary position in the production of the disease.

[1] Practitioner, Dec., 1901.

